# PAK4 phosphorylates cyclin-dependent kinase 2 to promote the G_1_/S transition during adipogenesis

**DOI:** 10.1038/s12276-025-01525-x

**Published:** 2025-09-30

**Authors:** Hwang Chan Yu, Su Hyeon Park, Hye Jin Jo, Hyunchae Sim, Mi Rin Lee, Gahee Kim, So-Young Park, Yoonji Lee, Eun Ju Bae, Byung-Hyun Park

**Affiliations:** 1 Graduate School of Medical Science and Engineering, Korea Advanced Institute of Science and Technology, Daejeon, Republic of Korea; 2 School of Pharmacy and Institute of New Drug Development, Jeonbuk National University, Jeonju, Republic of Korea; 3 School of Pharmacy, Sungkyunkwan University, Suwon, Republic of Korea; 4 Department of Surgery, Jeonbuk National University Hospital, Jeonju, Republic of Korea; 5 College of Pharmacy, Chung-Ang University, Seoul, Republic of Korea; 6 Department of Physiology, College of Medicine, Yeungnam University, Daegu, Republic of Korea

**Keywords:** Animal disease models, Obesity

## Abstract

p21-activated kinase 4 (PAK4), a member of the PAK family (PAK1–6), was initially recognized for its role in tumor development. Recently, we discovered PAK4’s involvement in triacylglycerol lipolysis in adipocytes. However, its function in adipogenesis remains unclear. Here we show that PAK4 plays a critical role in adipocyte differentiation. Following adipogenic stimulation, PAK4 protein levels increased. Knockdown of PAK4 in 3T3-L1 preadipocytes or human stromal vascular cells, as well as pharmacological inhibition of PAK4 in 3T3-L1 cells, impaired adipogenesis, as indicated by reduced expression of adipocyte marker genes and decreased lipid accumulation. Mechanistically, PAK4 phosphorylated cyclin-dependent kinase 2 at serine 106, a critical step for CCAAT/enhancer-binding protein β expression during mitotic clonal expansion. Consistent with these findings, preadipocyte-specific *Pak4*-knockout mice exhibited reduced fat mass and smaller adipocytes. These results reveal PAK4 as a crucial regulator of adipogenesis and, together with its inhibitory role in triacylglycerol lipolysis, further underscore its potential as a therapeutic target for obesity treatment.

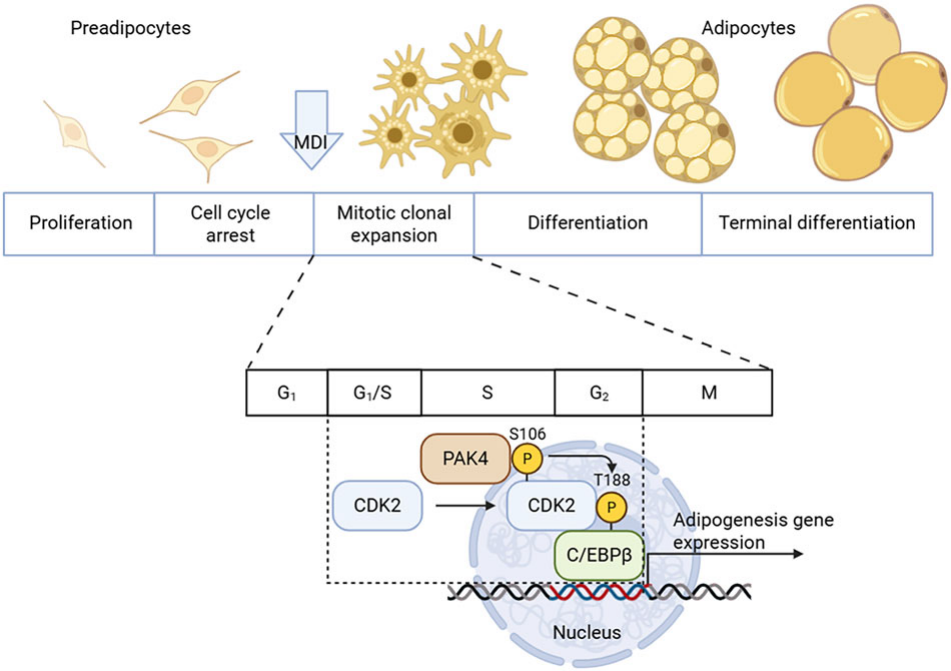

## PAK4 regulates adipogenesis in fat cells

Adipogenesis is the process by which stem cells become fat cells, and it is crucial for understanding obesity. Researchers identified a gap in understanding how the protein PAK4 affects this process. The study explored PAK4’s role in fat cell development and used human fat samples and mice to study this. They generated mice with specific genes turned off to examine the impact of PAK4 loss on fat development. They also used special techniques to look at proteins and their interactions. Researchers discovered that PAK4 helps another protein, CDK2, work better by adding a phosphate group to it. When they blocked PAK4, fat cell formation was reduced. This suggests that PAK4 is crucial for turning stem cells into fat cells. The study concludes that targeting PAK4 could be a new way to treat obesity by controlling how fat cells form.

This summary was initially drafted using artificial intelligence, then revised and fact-checked by the author.

## Introduction

Adipogenesis is the process by which mesenchymal stem cells differentiate into preadipocytes, which then mature into adipocytes. The early stage of adipogenesis involves cell cycle growth arrest, followed by the reentry of preadipocytes into the cell cycle, leading to mitotic clonal expansion^1^. During the initial phases of differentiation, the expression of CCAAT/enhancer binding proteins delta (C/EBPδ) and beta (C/EBPβ) increases in response to hormonal signals^2^. The final stage of adipogenesis is regulated by the coordinated expression of peroxisome proliferator-activated receptor gamma (PPARγ) and C/EBPα, ultimately resulting in the formation of mature adipocytes^3^.

Cyclin-dependent kinases (CDKs) are enzymes that regulate cell division by forming complexes with cyclins. Most tissues remain in the G_0_ phase, which can either progress to the next stage or undergo permanent arrest. Quiescent cells require stimulation, such as insulin, dexamethasone and agents that elevate cellular cAMP, to trigger intracellular signaling cascades, leading to CDK-mediated progression through the cell cycle^4^. In response to these signals, CDK–cyclin complexes phosphorylate retinoblastoma protein (RB), relieving its suppression of gene expression and enabling cell cycle advancement^5^.

Preadipocytes in the G_1_ phase express C/EBPβ, initiating a cascade of transcriptional events. The activation of C/EBPβ as a transcription factor involves a complex series of regulatory steps, including interactions with other proteins and posttranslational modifications. Typically, C/EBPβ is found in a repressed state and becomes activated through phosphorylation of a repressor domain situated between the N-terminal transactivation domains and the C-terminal bZIP region^6^. Its phosphorylation occurs sequentially, first by ERK at T188 (T235 in humans), followed later by GSK-3β at T179 (T226 in humans) or S184 (S231 in humans)^7^. In addition, various kinases, such as protein kinase A (S299/S288 in humans)^8^, homeodomain-interacting protein kinase 2 (T188)^9^, CDK2 (T188)^10^, protein kinase G (S176/S223 in humans)^11^, ribosomal S6 kinase-2 (T217/T266 in humans)^12^ and Ca^2+^-calmodulin-dependent kinase II (S276/S325 in humans)^13^ also phosphorylate C/EBPβ. Overall, phosphorylation of C/EBPβ enhances its DNA-binding activity.

p21-activated kinases (PAKs) are a group of serine/threonine kinases involved in regulating key cellular functions, such as cell survival, proliferation, apoptosis inhibition and cell morphology^14^. Despite sharing about 50% sequence similarity, group I (PAK1–3) and group II (PAK4–6) differ in other domains, affecting their role in promoting cell cycle progression and proliferation. PAK4, in particular, is recognized as an oncoprotein, implicated in cancers such as breast cancer^15^, non-small cell lung cancer^16^ and prostate cancer^17^. Beyond its role in cancer, our previous work has linked PAK4 to several biological processes, including myogenesis following muscle injury^18^, oxidative stress during ischemia–reperfusion injury^19,20^, ketone body production during fasting^21^, and insulin-stimulated glucose uptake in skeletal muscle^22^. Recently, we showed that PAK4 inhibits lipolysis in adipocytes, potentially contributing to obesity^23^. However, its role in adipogenesis remains unclear. Given PAK4’s links to cell cycle regulation and obesity, we hypothesized it may promote mitotic clonal expansion during adipogenesis.

## Materials and methods

### Human tissue samples

We obtained abdominal fat tissues located near the gallbladder from patients who underwent elective or emergency laparoscopic cholecystectomy at the Surgery Unit of Jeonbuk National University Hospital (Jeonju, Korea). All patients provided written informed consent, and the study received approval from the Institutional Review Board of Jeonbuk National University Hospital (permit no. JUH 2022-04-033).

### Animals

*Pak4*^*flox/flox*^ mice (B6.129S2-*Pak4*^tm2.1Amin^/J) and *Pdgfrb-Cre* mice (B6. *Pdgrfb-P2A-CreER*^*T2*^) were obtained from the Jackson Laboratory. Preadipocyte-specific *Pak4*-knockout (KO) mice (*Pak4*^*flox/flox*^;*Pdgfrb-Cre*) were generated by crossing *Pak4*^*flox/flox*^ and *Pdgfrb-Cre* mice. Genotyping was carried out using tail tips that were incubated with STE buffer (0.2% SDS, 100 mM Tris, 5 mM EDTA and 200 mM NaCl, pH 7.4) along with 0.5 mg/ml proteinase K for 12 h at 56 °C. Subsequently, a two-step PCR was conducted with Taq polymerase (Clontech) using specific forward (5′-GATGCAACGAGTGATGAG-3′) and reverse (5′-TCGGCTATACGTAACAGG-3′) primers, and the presence of a 496-bp band confirmed the *Pak4* genotype. All experimental mice were housed in a controlled barrier facility (12-h light/dark cycle, 23 ± 1 °C, 60–70% humidity). This study protocol was approved by the Institutional Animal Care and Use Committee of Jeonbuk National University Hospital (permit no. JBUH-2020-12-1).

### Body fat percentage

Percentage body fat was determined using a Bruker Minispec mq 7.5 NMR analyzer (Bruker Optics) as described previously^24^.

### Histology

Epidydimal adipose tissue (EAT) was promptly immersed in a 10% formalin solution in 0.1 M phosphate-buffered saline (PBS) for fixation. Subsequently, 6-μm-thick paraffin sections were prepared, blocked with 5% goat serum at room temperature for 40 min and stained with the primary antibody against perilipin-1 (#9349, Cell Signaling Technology) overnight at 4 °C. After washing with PBS, secondary antibody (#11001, Alexa Fluor 488-conjugated goat anti-mouse IgG1, Thermo Fisher Scientific) was incubated for 90 min at room temperature. Sections were then counterstained with 4′,6-diamidino-2-phenylindole (DAPI). The resulting images were visualized using an LSM510 confocal laser-scanning microscope (Carl Zeiss).

### Isolation of preadipocytes

EAT were aseptically collected from 8-week-old wild-type (WT) and *Pak4*-KO mice, finely minced and digested with 0.1% (w/v) type I collagenase (#LS004194, Worthington) at 37 °C for 30–45 min with gentle shaking. Digestion was halted by adding an equal volume of Dulbecco’s modified Eagle medium (DMEM) supplemented with 10% fetal bovine serum (FBS). The cell suspension was then filtered through a 100-μm mesh and centrifuged at 200*g* for 5 min. The resulting stromal vascular fraction was treated with ACK lysis buffer (Invitrogen) for 3 min to remove erythrocytes, followed by an additional centrifugation at 200*g* for 5 min. Preadipocytes were subsequently isolated from the stromal vascular fraction using a magnetic-activated cell sorting separation column positioned in a magnetic stand (#130-118-457, Miltenyi Biotec).

### Adipocyte differentiation of 3T3-L1 preadipocytes and stromal vascular cells

3T3-L1 murine preadipocytes were obtained from the American Type Culture Collection. Stromal vascular cells (SVCs) were isolated from EAT of mice or mesenteric fat tissues from humans and plated onto collagen-coated dishes until reaching confluency. The cells were cultured in DMEM supplemented with 10% FBS, penicillin (100 units/ml) and streptomycin (100 mg/ml) in a 10% CO_2_ incubator. To induce differentiation, an adipogenic cocktail (Methylxanthine (isobutyl-methylxanthine), Dexamethasone, and Insulin (MDI), including 10 μg/ml insulin, 1 μM dexamethasone and 0.5 mM isobutyl-methylxanthine) was added to the DMEM–10% FBS medium. After 2 days, the medium was replaced with DMEM–10% FBS containing 10 μg/ml insulin for an additional 2 days, followed by replacing the medium with DMEM–10% FBS every 2 days for another 4-day period.

### Transient transfection

In the knockdown experiment using SVCs, cells were electroporated with small interfering RNA (siRNA) targeting PAK4 (#93759, Bioneer) at 300 mV for 30 ms using a Microporator (Invitrogen). After electroporation, cells were cultured overnight in growth medium without antibiotics. For knockdown experiments in 3T3-L1 preadipocytes, siRNA was delivered using Lipofectamine RNAiMAX (Invitrogen). To overexpress CDK2 or its mutants, 1.5 μg of the respective plasmids were transfected into 3T3-L1 cells on day −1 using Lipofectamine RNAiMAX (Invitrogen). For transcriptional regulation assays of C/EBPβ, 1 μg of luciferase reporter vectors (#336841 CCS-001L, Qiagen) was transfected into 3T3-L1 cells on day −1.

### Oil Red O staining

Differentiated cells were fixed in 10% formalin for 10 min at room temperature. After fixation, the cells were washed with deionized water twice and incubated in 60% isopropanol for 5 min. Cells were completely air dried at room temperature before Oil Red O working solution (2 g/l Oil Red O in 60% isopropanol) was applied. After incubation at room temperature for 10 min, the Oil Red O solution was removed and the cells were washed with deionized water five times before the images were acquired for analysis.

### Flow cytometry

3T3-L1 preadipocytes were induced to differentiate into adipocytes for 24 h, then collected, washed with PBS and fixed overnight in 70% precooled ethanol at −20 °C. After washing with PBS, the cells were resuspended in 500 μl of PBS containing 100 μg/ml RNase1 and incubated for 30 min at room temperature. They were then stained with propidium iodide solution (Roche) at a final concentration of 50 μg/ml for 30 min at 4 °C. The stained cells were analyzed using a Accuri C6 flow cytometer (BD Biosciences).

### Western blotting and co-immunoprecipitation (co-IP)

Plasma membrane and cytoplasmic fractions were prepared using the MEM-PER membrane protein extraction kit (#89842, Thermo Fisher Scientific). Tissue homogenates or cell lysates (20 μg) were separated by 6–14% SDS–PAGE and transferred to polyvinylidene difluoride membranes. After blocking with 5% skim milk, blots were probed with primary antibodies against PAK4 (#G222), p-PAK4-S474 (#3241), C/EBPα (#2295), C/EBPβ (#3082), FAS (#3189), FABP4 (#2120), PLIN1 (#9349), cyclin D1 (#2922), SMYD3 (#12859), p-CDK2-T160 (#2561), p-CDK4 (#5884), RB (#9302), p-RB (#9308), lamin B1 (#13435), β-tubulin (#2146, all from Cell Signaling Technology), MAPK6 (#ab53277), PCNA (#ab18197), p-C/EBPβ (#ab52194, all from Abcam), ACC (#sc-137104), PPARγ (#sc-7273), CDK2 (#sc-6248), Ub (#sc-166553, all from Santa Cruz Biochemicals) or HSP90 (#ADI-SPA-846, Enzo Life Science). An affinity-purified rabbit monoclonal antibody targeting p-CDK2-S106 was generated against a peptide corresponding to the sequence GIPLPLIKS(106)YLFQLLQ in mouse and human CDK2 by GWVitek.

For co-IP, 100 μg protein was incubated with anti-CDK2 (#sc-6248, Santa Cruz Biochemicals), anti-C/EBPβ (#3082) or anti-Myc (#9402, both from Cell Signaling Technology) overnight at 4 °C, followed by protein G agarose (#15920-010, Invitrogen) for 2 h at 4 °C. Blots were probed with antibodies against C/EBPβ, p-C/EBPβ, PCNA, p-PAK4, PAK4, p-CDK2-T160, CDK2, p-CDK2-S106 or ubiquitin. Immunoreactive bands were detected with a Las-4000 imager (GE Healthcare Life Science).

### RNA isolation and qPCR

Total RNA was isolated from 3T3-L1 adipocytes using the RNA Iso kit (TaKaRa). The RNA was precipitated with isopropanol, dried with 70% ethanol and dissolved in diethyl pyrocarbonate-treated distilled water. First-strand cDNA was synthesized using random hexamer primers from the first-strand cDNA synthesis kit (Applied Biosystems). Specific primers for the target genes were designed using PrimerBank (https://pga.mgh.harvard.edu/primerbank). The oligonucleotide primers for qPCR were as follows: *Pak4* forward 5′-GCTCCCCTTTGAAGATGTCA-3′, reverse 5′-GACCCACAAGGACTCAAGGA-3′; *Gapdh* forward 5′-CGTCCCGTAGACAAAATGGT-3′, reverse 5′-TTGATGGCAACAATCTCCAC-3′. qPCR reactions were performed in a final volume of 10 µl, containing 10 ng of reverse-transcribed total RNA, 200 nM of both forward and reverse primers, and PCR master mix. The qPCR analysis was conducted in 384-well plates using the ABI Prism 7900HT Sequence Detection System (Applied Biosystems).

### Proximity ligation assay

Protein interactions were assessed using a Duolink proximity ligation assay (PLA) kit (#DUO92002, Sigma-Aldrich) as previously described^23^. In brief, 3T3-L1 adipocytes were fixed with 10% neutral buffered formalin, permeabilized with PBS/0.1% Triton X-100 and incubated with anti-PAK4 (#sc-390507, Santa Cruz Biochemicals) in conjunction with anti-CDK2 (#7882, Cell Signaling Technology). Samples were then incubated with Duolink in situ PLA Probes (anti-rabbit Minus and anti-mouse Plus) for 1 h. Signals were amplified with polymerase using In Situ Detection Reagents Green. Finally, cells were counterstained with DAPI and images were captured using an LSM880 confocal laser scanning microscope (Carl Zeiss).

### In vitro kinase assay

Recombinant human CDK2 (3 μg, #ab268339 Abcam) was incubated with recombinant human PAK4 protein (1 μg, #ab96405, Abcam) in assay buffer (50 mM Tris-HCl, 10 mM MgCl_2_, 2 mM dithiothreitol and 0.1 mM EDTA, pH 7. 6) containing 5 μCi [γ-^32^P] ATP and 50 μM cold ATP at 30 °C for 10 min. The reaction mixtures were then subjected to SDS–PAGE, and ^32^P-labeled proteins were detected by autoradiography. For Coomassie blue staining, the gel was stained for 1 h in protein staining buffer (#ab119211, Abcam).

### Mutagenesis

Mutant constructs of the human CDK2 plasmid vector (#RC200494, Origene) were generated using a site-directed mutagenesis kit (#EZ004S, Enzynomics). Specifically, the CDK2^S106A^, CDK2^S106D^ and CDK2^T160A^ variants were created by introducing point mutations that substituted the serine residue at position 106 with alanine or aspartic acid, and the threonine residue at position 160 with alanine. The corresponding nucleotide changes were: CDK2^S106A^ (AGC → GCC), CDK2^S106D^ (AGC → GAC) and CDK2^T160A^ (ACU → GCU).

### Global phosphoproteomic and LC–MS/MS analysis

To identify the phosphorylated substrates by PAK4, 3T3-L1 preadipocytes were transfected with 1 μg of Flag-PAK4 (#HG12175, Sino-Biological). After 1 day of differentiation, the cells were lysed in T-PER buffer containing protein phosphatase inhibitors. Cell lysate (500 μg) was subjected to SDS–PAGE and visualized by colloidal Coomassie blue staining. After going through the in-gel digestion process, they were analyzed by liquid chromatography–tandem mass spectrometry (LC–MS/MS) as described previously^21^. Potential proteins of interest were defined as phosphotryptic peptides that exhibited (1) a negative or positive change of >2-fold in the phosphorylation state (phosphoprotein/protein) and (2) a change with *P* < 0.05 across the three replicates.

### Prediction of PAK4–CDK2 complex model

Computational simulation was conducted to predict the complex model of PAK4 and CDK2. The X-ray crystal structures of human PAK4 (PDB ID: 4XBR)^25^ and CDK2 (PDB ID: 1W98)^26^ were retrieved from Protein Data Bank (PDB). Among the available X-ray crystal structures of CDK2, the one in which the C-terminal loop does not hinder the location of S106 was selected. Protein–protein docking was conducted using the PIPER module implemented in Schrödinger with the attraction restraints between S474 of PAK4 and S106 of CDK2. The ten most populated models were determined through clustering analysis and analyzed to identify the most biologically relevant interactions by assessing the docking scores and visually inspecting the interface. The final docking model was further minimized using the Desmond module implemented in Schrödinger to validate the stability and feasibility of the predicted protein–protein interactions. The molecular graphic figure was created using PyMOL v.2.5.4 (Schrödinger).

### Statistics and reproducibility

All experiments requiring statistical analysis were performed at least three times with similar results. Data are expressed as the mean ± standard deviation (s.d.). Data from animal and cell studies were collected in a randomized and blinded fashion, and no data were excluded during the statistical analysis. Data distribution was assumed to be normal, but this was not formally tested.

For comparisons between two groups, Student’s unpaired *t*-test was used. The Pearson correlation coefficients were calculated for continuous variables. A *P* value less than 0.05 was considered significant. We utilized GraphPad 9.5 software for statistical analysis.

## Results

### PAK4 knockdown suppresses adipogenesis

**Fig. 1:**
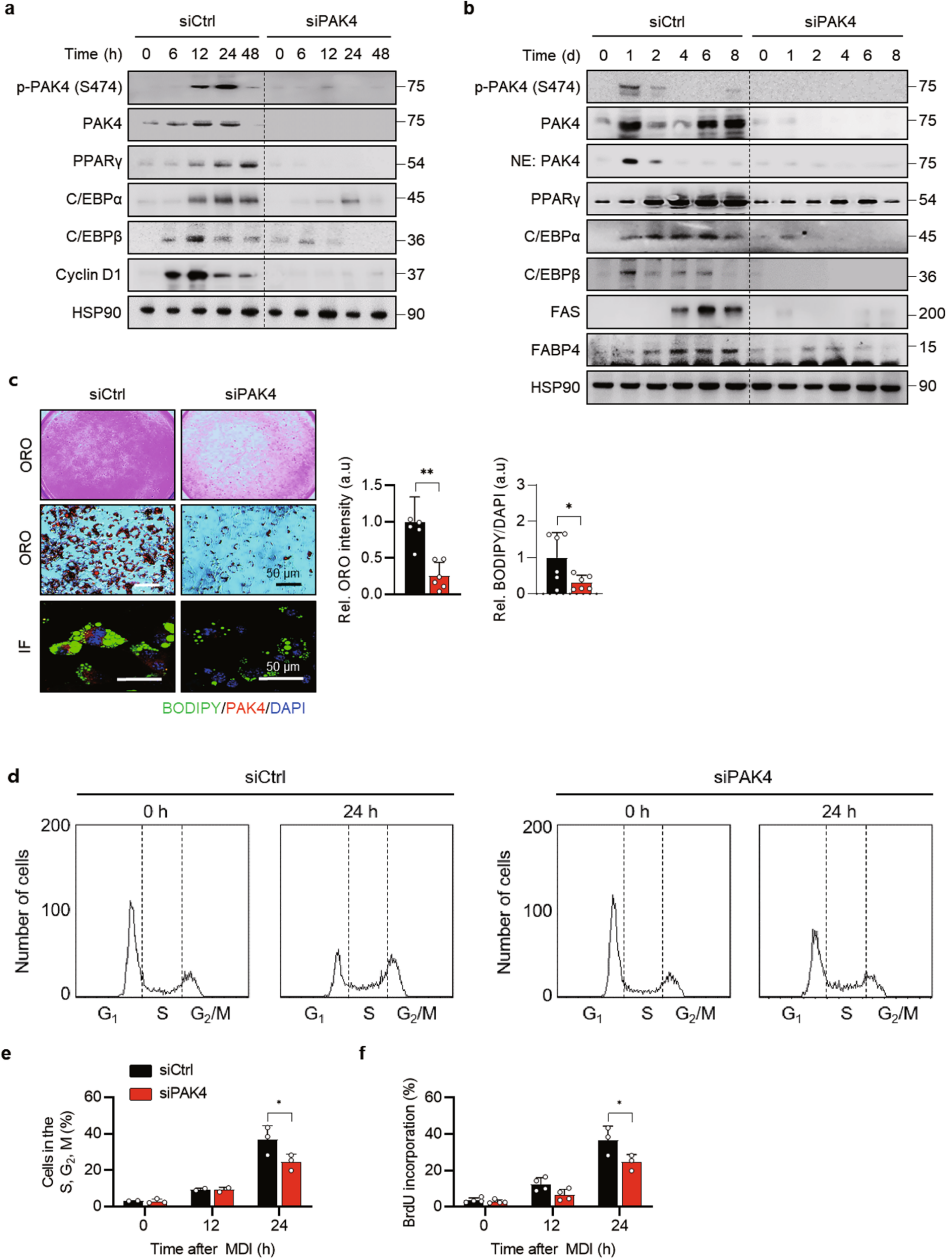
Inhibition of mitotic clonal expansion by PAK4 knockdown. **a**, **b** 3T3-L1 preadipocytes were transfected with either scrambled siRNA (siCtrl) or PAK4-targeting siRNA (siPAK4). Twelve hours after siRNA transfection, cells were induced to differentiate for the indicated durations: up to 48 h (**a**) or 8 days (**b**). Adipogenic marker gene expression was assessed via western blotting. **c** After 8 days of differentiation, 3T3-L1 cells were stained with Oil Red O (ORO) or immunostained using BODIPY. **d** The cell cycle distribution of 3T3-L1 preadipocytes was analyzed by flow cytometry after 24 h of MDI treatment. **e**, **f** After 12 or 24 h of MDI treatment, the number of cells in the S, G_2_ and M phases (**e**) and BrdU incorporation (**f**) were measured. Values are mean ± s.d. ^*^P < 0.05, ^**^P < 0.01.

To determine whether PAK4 plays a role in adipogenesis, we examined its expression after treatment with MDI. Both PAK4 protein and mRNA levels significantly increased on day 1, declined by day 4 and then rose again (Supplementary Fig. 1a,b), suggesting its involvement in the adipogenic process. In PAK4-knockdown cells, the expression of early and late adipogenic markers was markedly reduced (Fig. 1a). As a result, adipogenesis was impaired, shown by reduced expression of fatty acid synthase (FAS) and fatty acid binding protein 4 (FABP4), and diminished lipid accumulation as observed by Oil Red O staining and boron-dipyrromethene (BODIPY) immunostaining (Fig. 1b, c). Given the interaction between PAK4 and cyclin D1 in ovarian cancer^27^ and cyclin D1’s role in the G_1_/S phase^28^, we found that cyclin D1 was reduced by PAK4 knockdown (Fig. 1a), leading to G_1_/S cell cycle arrest. Flow cytometry and bromodeoxyuridine (BrdU) incorporation assays confirmed this arrest (Fig. 1d–f). These findings suggest that PAK4 deficiency hinders adipogenesis by arresting the cell cycle at the G_1_/S phase.

### PAK4 inhibitor impairs adipogenesis

**Fig. 2:**
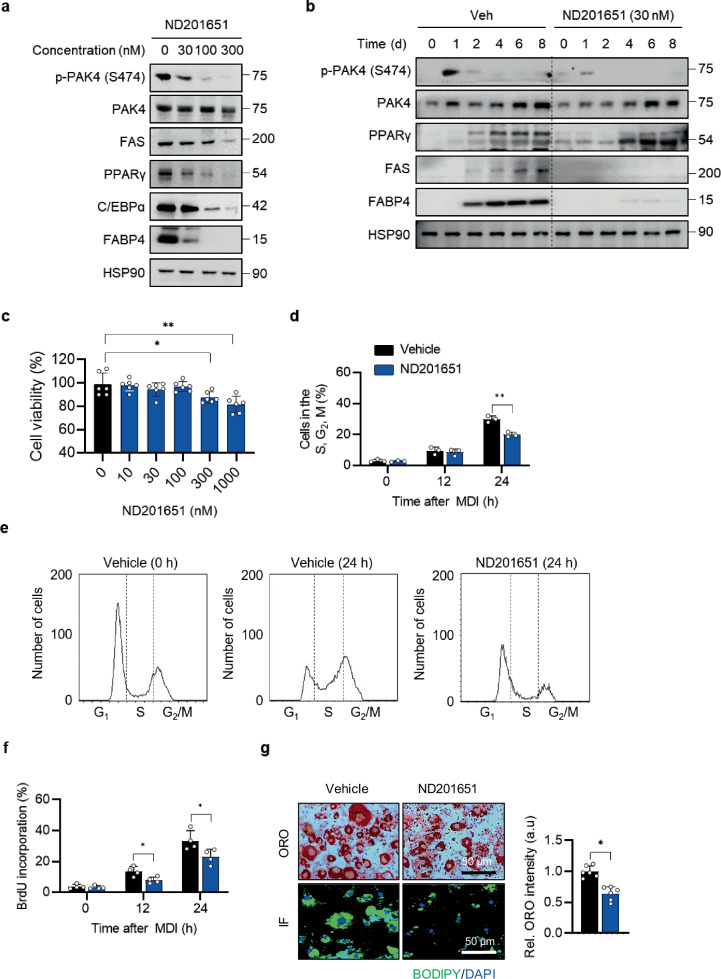
Inhibition of adipogenesis by the PAK4 inhibitor ND201651. **a** 3T3-L1 preadipocytes were treated with varying concentrations of ND201651, followed by differentiation induction with MDI. All adipogenic marker proteins were analyzed on day 8 post-MDI treatment, while PAK4 phosphorylation was assessed on day 1. **b** Temporal changes in adipogenic marker expression in the presence or absence of ND201651 (30 nM). **c** 3T3-L1 preadipocytes were treated with different concentrations of ND201651 for 2 days, and cell viability was measured using the MTT assay (*n* = 6). **d**–**f** 3T3-L1 preadipocytes were treated with MDI for 12 or 24 h with or without 30 nM ND201651. Flow cytometry was performed to analyze cell cycle distribution (**d**), quantify the number of cells in the S, G_2_ and M phases (**e**, *n* = 4) and determine BrdU incorporation (**f**, *n* = 4). **g**, After 8 days of differentiation, representative images were captured from each group following Oil Red O (ORO) staining or BODIPY immunostaining to assess lipid accumulation. ORO intensity was quantified with spectrophotometer at 540 nm wavelength after dissolving in 70% isopropanol (*n* = 5). Values are mean ± s.d. ^*^P < 0.05, ^**^P < 0.01.

We further explored the effect of the PAK4 inhibitor, ND201651^20^, on adipogenesis in 3T3-L1 cells. Similar to the effects seen with PAK4 knockdown, the inhibitor suppressed adipogenesis in a dose- and time-dependent manner, without cytotoxicity up to concentrations of 100 nM (Fig. 2a–c). This was further confirmed by cell cycle analysis, BrdU incorporation, Oil Red O staining and BODIPY immunostaining (Fig. 2d–g). In addition, the PAK4 inhibitor PF-3758309^29^ produced similar results (Supplementary Fig. 2a–e).

### PAK4 phosphorylates CDK2 at S106

**Fig. 3:**
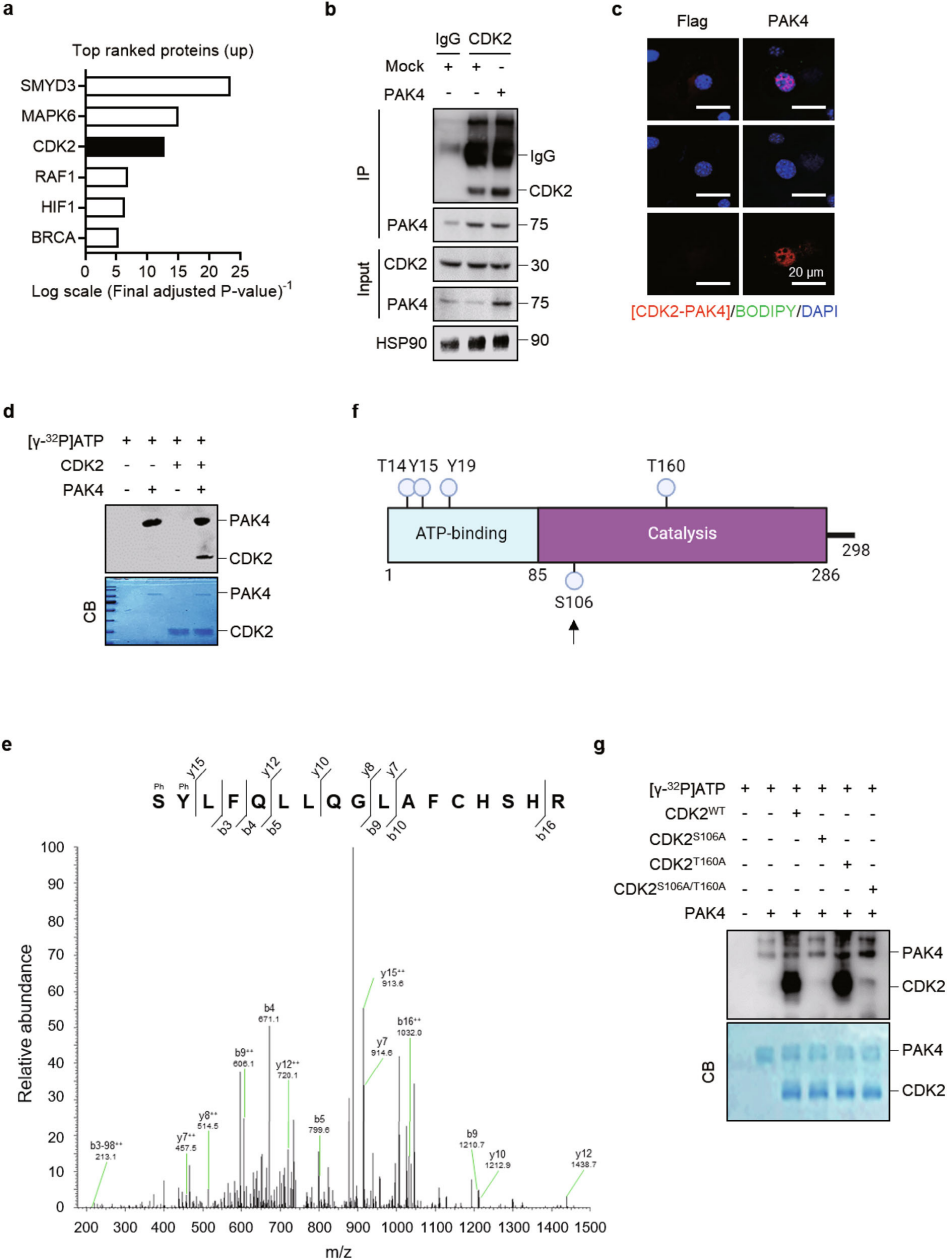
Direct phosphorylation of CDK2 by PAK4. **a** After transfection of 3T3-L1 preadipocytes with Flag-PAK4, whole-cell lysates were analyzed using two-dimensional gel electrophoresis, LC–MS/MS for phosphotryptic peptide identification and quantification. The top-ranked phosphorylated proteins are listed. **b** Co-IP was performed on whole-cell lysates to assess PAK4’s interaction with CDK2. **c** PLA was performed in 3T3-L1 preadipocytes to determine PAK4’s interaction with CDK2. **d** In vitro phosphorylation of CDK2 by PAK4 was evaluated by incubating recombinant CDK2 and PAK4 with [γ-^32^P]ATP, followed by autoradiography and Coomassie blue (CB) staining. **e** 3T3-L1 preadipocytes were transfected with PAK4, and CDK2 phosphorylation was analyzed via LC–MS/MS following in-gel digestion. A representative spectrum is shown. **f** The domain structure of human CDK2 protein is illustrated, showing multiple phosphorylation sites. The arrow indicates phosphorylation site by PAK4. **g** 3T3-L1 preadipocytes were transfected with either CDK2^WT^, CDK2^S106A^, CDK2^T160A^ or CDK2^S106A/T160A^. Phosphorylation of CDK2 immunoprecipitates (30 μg) by PAK4 was assessed by incubation with [γ-^32^P]ATP, followed by autoradiography and CB staining.

To identify downstream substrates of PAK4 kinase, we conducted a phosphoproteomic analysis comparing preadipocytes overexpressing PAK4 with control preadipocytes. The primary screening focused on differences in phosphoprotein/total protein levels between the two groups. Based on the criteria outlined in the Methods section, the top three candidates were SMYD3, MAPK6 and CDK2 (Fig. 3a). SMYD3 and MAPK6 were excluded from further analysis as they were not detected in PAK4 immunoprecipitates (Supplementary Fig. 3a,b); however, CDK2 was present in PAK4 immunoprecipitates (Fig. 3b). We therefore focused on analyzing PAK4’s regulation of CDK2.

The direct interaction between PAK4 and CDK2 was confirmed using a PLA (Fig. 3c). A cell-free phosphorylation assay with recombinant human CDK2 showed that PAK4 enhances CDK2 phosphorylation (Fig. 3d). LC–MS/MS analysis further identified S106 (corresponding to S106 in human) within the catalytic domain of CDK2 as the phosphorylation site targeted by PAK4 (Fig. 3e,f).

To validate CDK2 phosphorylation at S106, we generated the CDK2^S106A^ (serine-to-alanine substitution at S106), the CDK2^T160A^ (threonine-to-alanine substitution at T160) and the CDK2^S106A^/^T160A^ double mutant. These constructs were transfected into 3T3-L1 cells with or without PAK4. In vitro phosphorylation assays revealed that PAK4 phosphorylated CDK2 in the presence of the CDK2^T160A^ mutant, but not when CDK2^S106A^ or CDK2^S106A/T160A^ was expressed (Fig. 3g), confirming S106 as the specific phosphorylation site targeted by PAK4.

**Fig. 4:**
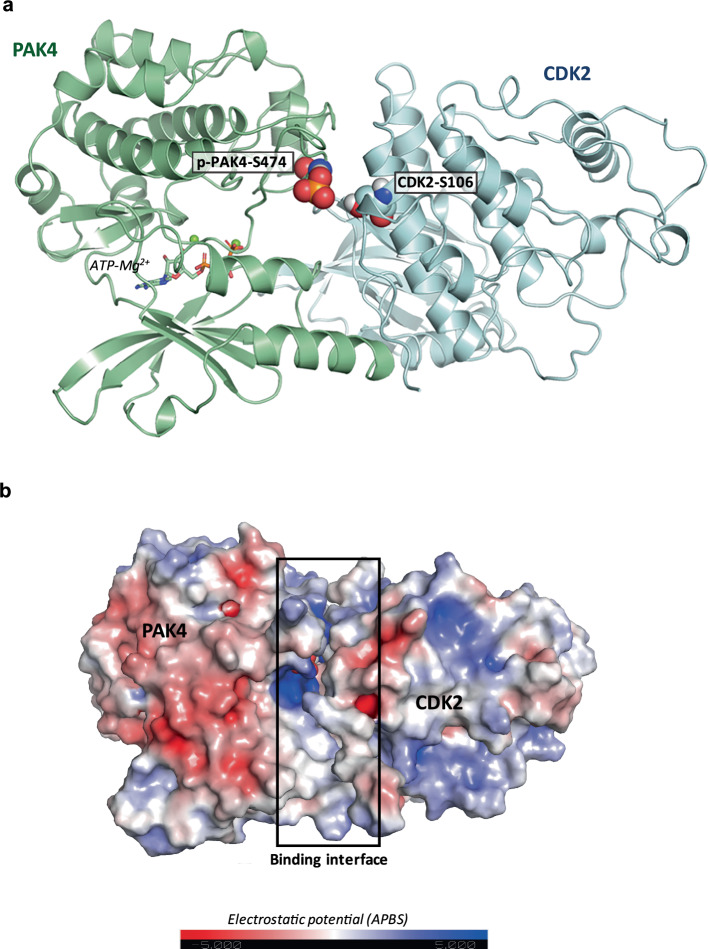
A predicted model of the interaction between PAK4 and CDK2. **a** The binding interaction between PAK4 and CDK2 was predicted through computational modeling. The secondary structures of PAK4 and CDK2 are shown in pale-green and light-blue ribbons, respectively. The two residues involved in phosphorylation, that is, PAK4-pS474 and CDK2-S106, are depicted as spheres. The ATP-Mg^2+^ molecule is displayed as sticks at the ATP-binding site. **b** The molecular surfaces of PAK4 and CDK2 are displayed with electrostatic potential. The binding interface, outlined by a black box, illustrates the complementary electrostatic interactions between PAK4 (left) and CDK2 (right), with regions of positive charge in blue and negative charge in red. The model suggests that PAK4-mediated phosphorylation of CDK2 at the S106 residue is structurally feasible.

The protein–protein docking study predicted the structural complex of PAK4 and CDK2, identifying key residues involved in their interactions. Phosphorylated S474 of PAK4 and S106 of CDK2 are critical for their interaction, and the complex model showed the feasibility of the phosphorylation mechanism we have identified (Fig. 4a). The binding interface, marked by a black box (Fig. 4b), shows a substantial region of complementary surface and electrostatic interaction between PAK4 and CDK2. These findings provide a molecular basis for the regulatory role of PAK4 in CDK2 activity, highlighting its potential as a modulator in cell cycle control.

### PAK4 phosphorylation of CDK2 at S106 is required for adipogenesis

**Fig. 5:**
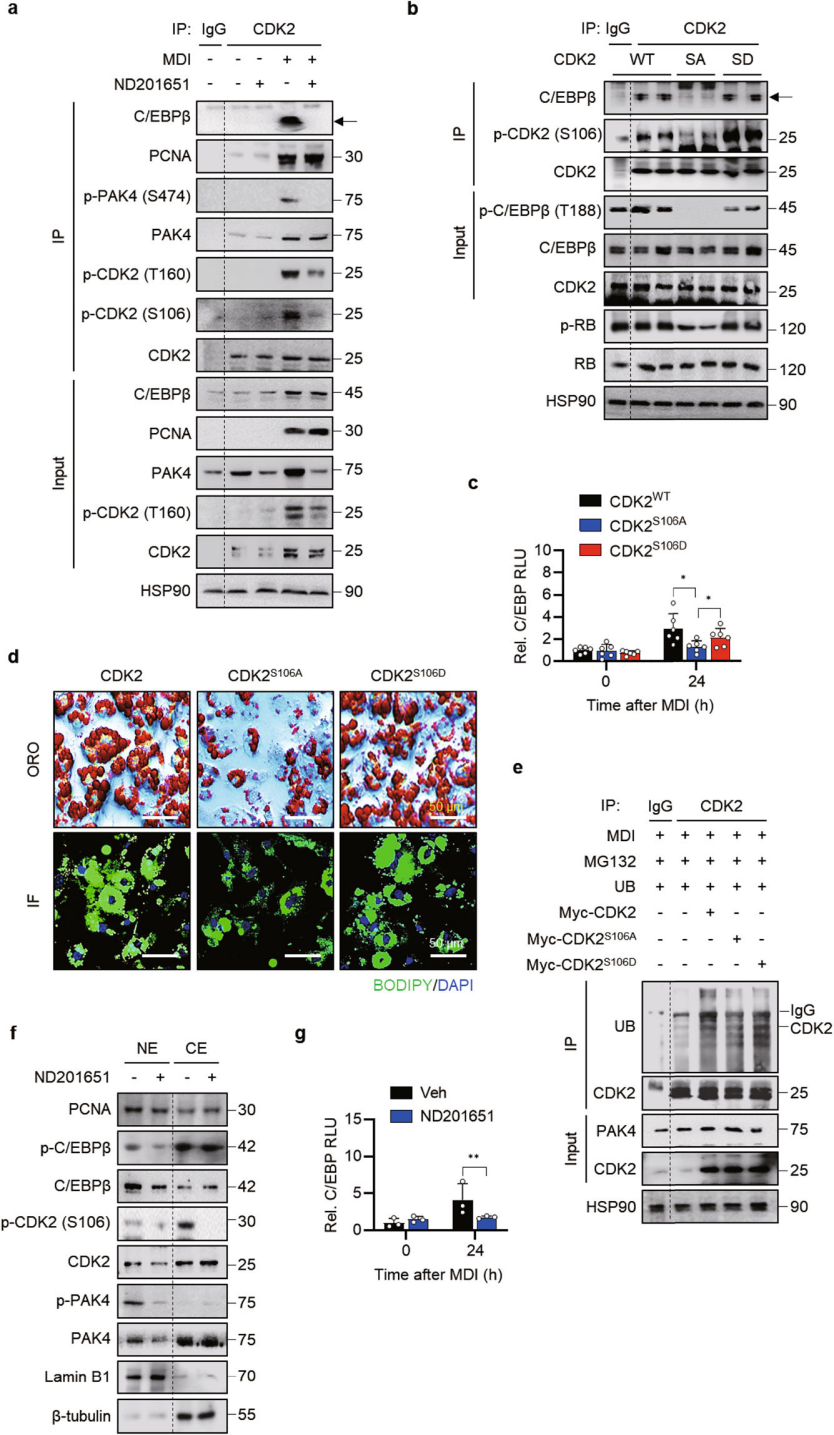
Essential role of PAK4-mediated phosphorylation of CDK2 in adipogenesis. **a** 3T3-L1 preadipocytes were pretreated with 30 nM ND201651 before MDI treatment. Co-IP was performed on day 1 to assess CDK2 phosphorylation and its interaction with C/EBPβ. **b**–**e** 3T3-L1 preadipocytes were transfected with either phosphodeficient CDK2 (CDK2^S106A^, SA) or phosphomimetic CDK2 (CDK2^S106D^, SD), then induced to differentiate with MDI treatment. After 24 h, C/EBPβ phosphorylation (**b**) and C/EBP-transcriptional activity (**c**, *n* = 3) were analyzed. **d** Lipid accumulation was assessed after 8 days of differentiation using Oil Red O staining and BODIPY immunostaining. **e** CDK2 ubiquitination was analyzed in CDK2 immunoprecipitates from cell lysates collected 24 h after the initiation of differentiation following transfection. **f** Nuclear translocation of CDK2 was examined by western blotting after 24 h of differentiation, with or without 30 nM ND201651 treatment. **g** 3T3-L1 preadipocytes were treated with 30 nM ND201651, followed by differentiation induction with MDI for 24 h. C/EBP-luciferase activity was then measured (*n* = 3). Veh, vehicle; RLU, relative luciferase unit; NE, nuclear extract; CE, cytosolic extract.. Values are mean ± s.d. ^*^P < 0.05, ^**^P < 0.01.

Because the CDK2–cyclin A complex induces phosphorylation of C/EBPβ at T188 and initiates mitotic clonal expansion^10^, we investigated whether PAK4-mediated phosphorylation of CDK2 at S106 could affect the effect of C/EBPβ on this process. Co-IP experiments on 3T3-L1 preadipocytes treated with MDI, with or without ND201651, demonstrated that C/EBPβ, CDK2 and PAK4 interact with each other (Fig. 5a). Interestingly, phosphorylation of CDK2 at T160 was diminished by ND201651, which mirrored the effect observed at the S106 phosphorylation site, suggesting a potential relationship between T160 and S106. However, PCNA, another CDK2 binding partner^30^, was not affected by ND201651, indicating specific action of PAK4 on CDK2.

To assess the functionality of PAK4-mediated phosphorylation of CDK2 at S106, we transfected 3T3-L1 cells with either a phosphodeficient mutant (CDK2^S106A^) or a phosphomimetic mutant (CDK2^S106D^) and evaluated their impact on C/EBPβ. Transfection with CDK2^S106A^ resulted in a reduced interaction between CDK2 and C/EBPβ (Fig. 5b) and decreased C/EBP transcriptional activity (Fig. 5c), whereas CDK2^S106D^ had no effect on these. Consistently, CDK2^S106A^ inhibited adipogenesis in 3T3-L1 cells (Fig. 5d), demonstrating the significance of CDK2 phosphorylation at S106 in the adipogenesis process. To rule out the possibility that this disruption was due to CDK2 protein degradation, treatment with the proteasome inhibitor MG132 confirmed that CDK2 levels remained stable regardless of phosphorylation status (Fig. 5e). Considering that active CDK2 and C/EBPβ are probably present in the nucleus, we isolated nuclear and cytosolic fractions from 3T3-L1 cells after ND201651 treatment. The results indicated that PAK4 inhibition led to a decrease in nuclear protein levels of both CDK2 and C/EBPβ (Fig. 5f) and transcriptional activity of C/EBP (Fig. 5g), suggesting that the PAK4–CDK2 axis functionally regulates C/EBPβ.

### Preadipocyte-specific Pak4 KO exhibits reduced fat mass

**Fig. 6:**
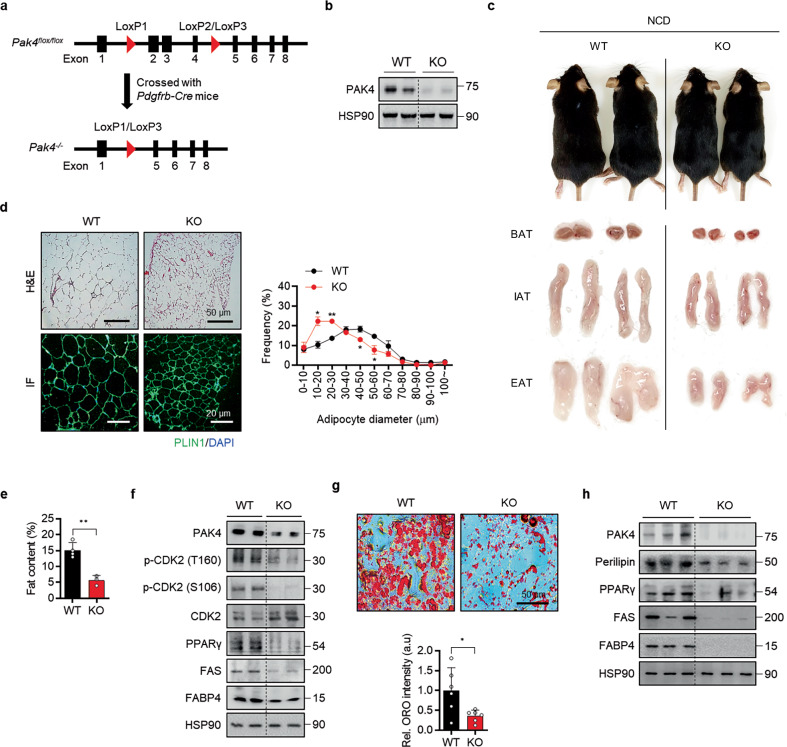
Metabolic phenotypes of preadipocyte-specific *Pak4*-KO mice under normal chow diet feeding. **a** A schematic representation of preadipocyte-specific *Pak4* ablation using *Pdgfrb-Cre* mice. **b** Western blot analysis of primary preadipocytes isolated from WT and *Pak4*-KO mice to assess PAK4 expression. **c** Representative images of *Pak4*-KO and WT littermates (top), along with gross morphology of brown adipose tissue (BAT), inguinal adipose tissue (IAT) and EAT (bottom) at 14 weeks of age. **d** Microscopic analysis of EAT was conducted using hematoxylin and eosin (H&E) staining and BODIPY immunostaining, with adipocyte size in the H&E sections quantified. **e** Fat content in mice was measured using an NMR analyzer. **f** PAK4-mediated phosphorylation of CDK2 and the expression of adipogenic markers were examined in EATs from both WT and *Pak4*-KO mice. **g**, **h** SVCs isolated from EAT of WT and *Pak4*-KO mice were differentiated into mature adipocytes for 8 days, and adipogenic marker protein levels and lipid accumulation were assessed. Values are mean ± s.d. ^*^P < 0.05, ^**^P < 0.01.

To establish tamoxifen-inducible preadipocyte-specific *Pak4*-KO mice (*Pak4*^*flox/flox*^;*Pdgfrb-Cre*), we crossed *Pdgfrb-Cre* mice^31^ and *Pak4*^*flox/flox*^ mice (Fig. 6a). The successful deletion of PAK4 in primary preadipocytes was validated by western blot analysis (Fig. 6b). The resulting *Pak4*-KO mice were born at the expected Mendelian ratio and appeared indistinguishable from their WT littermates. However, 3 months after tamoxifen treatment, the *Pak4*-KO mice exhibited smaller body size, reduced fat pads and smaller adipocyte sizes compared with their WT counterparts (Fig. 6c,d). This trend was also observed through body fat mass measurements using a nuclear magnetic resonance analyzer (Fig. 6e). Western blot analysis revealed a marked decrease in both CDK2 phosphorylation and the levels of adipogenic markers in EAT (Fig. 6f). Moreover, we isolated SVCs from the EAT and induced their differentiation into adipocytes, with Oil Red O staining and adipogenesis marker expression confirming the tissue analysis results (Fig. 6g,h).

### PAK4 knockdown reduces adipogenesis in human adipocytes

**Fig. 7:**
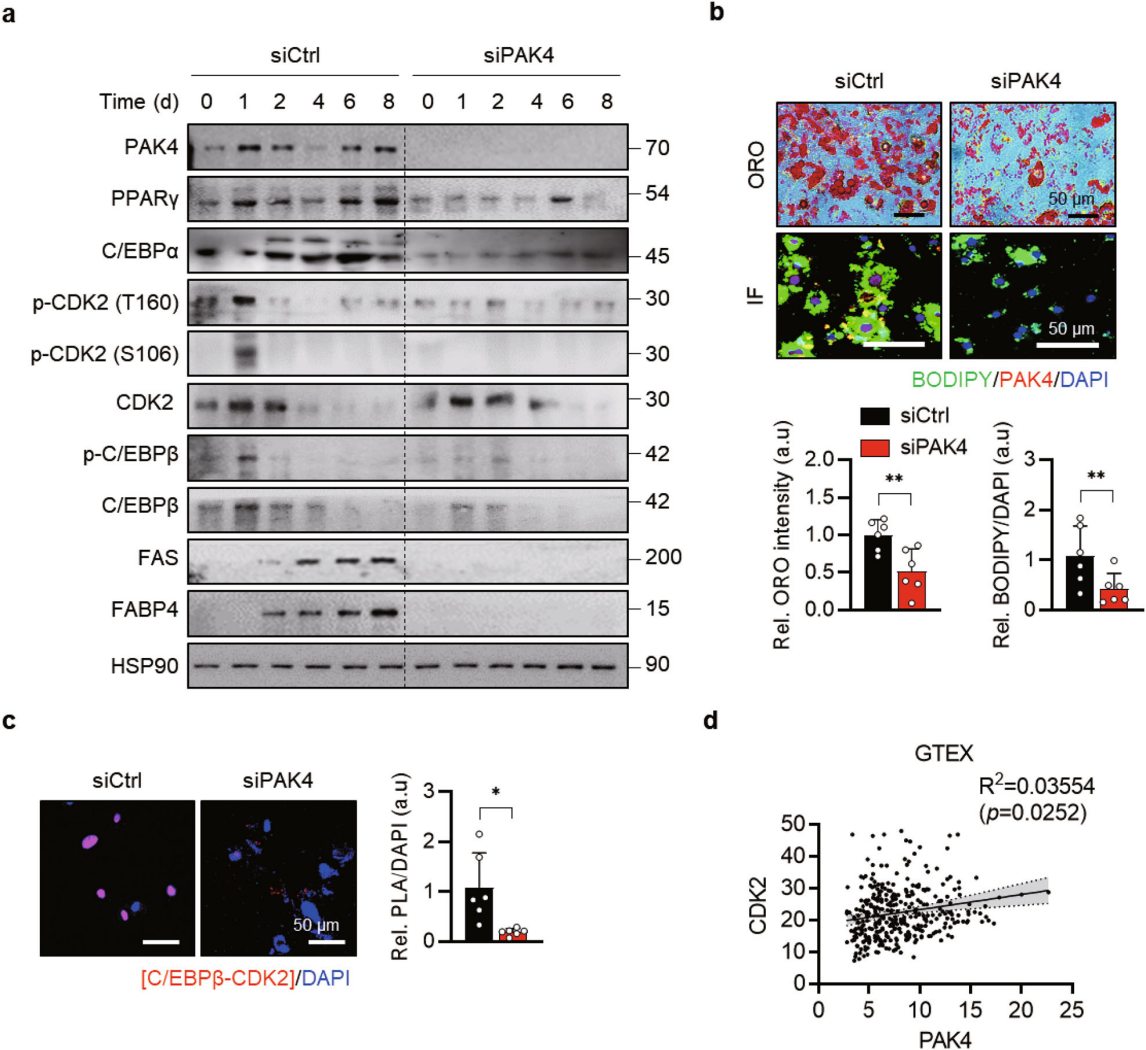
PAK4 interaction with CDK2 in human adipocytes. **a** SVCs isolated from mesenteric fat tissues of human subjects were transfected with PAK4 siRNA, followed by differentiation into mature adipocytes with MDI cocktail for the indicated durations. Protein levels of adipogenic markers were assessed via western blotting. **b** After 6 days of differentiation, Oil Red O staining and BODIPY immunostaining were conducted to evaluate lipid accumulation. **c** After 1 day of differentiation, the interaction between CDK2 and C/EBPβ was analyzed using PLA (*n* = 6 per group). **d** Genotype tissue expression (GTEx) analysis of human adipose tissues (*n* = 355).

Finally, we isolated SVCs from fat pads near the gallbladder of patients undergoing elective or emergency laparoscopic cholecystectomy. Consistent with the findings in adipocytes differentiated from 3T3-L1 preadipocytes and SVCs from *Pak4*-KO mice, PAK4 knockdown resulted in decreased protein levels of adipogenesis markers (Fig. 7a), reduced fat accumulation after MDI treatment (Fig. 7b) and weakened interaction between CDK2 and C/EBPβ (Fig. 7c). In addition, analysis of the human adipose tissue GTEx database showed a positive correlation between *PAK4* mRNA and *CDK2* mRNA levels (Fig. 7d). Together, these findings indicate that PAK4 in visceral fat promotes adipogenesis and may serve as a molecular marker of obesity in both mice and humans.

## Discussion

Adipogenesis is a hallmark of obesity, characterized by the progression of precursor cells into mature adipocytes. During mitotic clonal expansion, as the number of cells increases, signaling pathways related to the cell cycle are activated. Cyclin D and CDK4/CDK6 complexes regulate the early G_1_ phase, while cyclin E and CDK2 complexes are essential for the G_1_/S phase transition^5^. In this study, we demonstrate that PAK4 promotes adipogenesis in 3T3-L1 cells by modulating CDK2. Specifically, PAK4 binds to and phosphorylates CDK2 at S106, which is crucial for the interaction between CDK2 and C/EBPβ. This interaction allows cells arrested in the cell cycle to reenter and progress to the G_1_/S phase transition.

Several kinases, including protein kinase A, CDK2, protein kinase G, ribosomal S6 kinase-2 and Ca^2+^-calmodulin-dependent kinase II^8,11–13^, are known to phosphorylate C/EBPβ at various sites. Notably, the dual phosphorylation of C/EBPβ at T188 by ERK and T198 (or S184) by GSK3β is well established^7^, and CDK2 also phosphorylates C/EBPβ at T188^10^. Our study reveals that PAK4-mediated phosphorylation of CDK2 at S106 is critical for CDK2’s interaction with C/EBPβ, leading to phosphorylation of T188 and nuclear translocation of C/EBPβ and, ultimately, cell cycle progression in adipocytes. CDK2 activation requires its association with cyclin E^32^, removal of inhibitory phosphorylation on T14 and Y15^33^, and phosphorylation at T160 by ERK^34^. We propose that S106 phosphorylation by PAK4 is necessary for CDK2’s full activation. Interestingly, phosphorylation at CDK2’s T160 and S106 sites has distinct effects. T160 phosphorylation, which occurs in the T-loop, is crucial for CDK2’s nuclear translocation^34^, whereas S106 phosphorylation affects both the phosphorylation of T188 and nuclear translocation of C/EBPβ. Although it remains unclear whether S106 phosphorylation influences T160 or vice versa, our findings suggest that S106 phosphorylation promotes C/EBPβ’s nuclear translocation, while T160 phosphorylation enhances its transcriptional activity.

Many anticancer drugs target rapidly dividing cells, and because adipogenesis involves the proliferation and differentiation of preadipocytes, anticancer drugs such as doxorubicin, cisplatin and tamoxifen inhibit adipogenesis^35–37^, whereas some, such as imatinib, may promote it^38^. These anticancer drugs typically reduce the expression of critical adipogenic transcription factors, PPARγ and C/EBPα. In our study, we used PF-3758309 and ND201651 as PAK4 inhibitors. PF-3758309, developed by Pfizer as an anticancer drug for solid tumors^29^, was withdrawn from phase I trials owing to severe side effects and off-target effects. ND201651, created by our group, has shown antioxidative^19,20^, antisteatotic^21^, antiobesity^23^ and antidiabetic effects^22^. Like other anticancer agents, ND201651 inhibits adipogenesis but targets C/EBPβ. Despite differing mechanisms of action, the impact of these drugs on adipogenesis can lead to metabolic complications in patients with cancer, including changes in body fat distribution, weight loss and insulin resistance.

While our findings highlight the role of preadipocyte PAK4 in adipogenesis, several points should be noted. First, the optimal approach is to use Cre mice that target *Pdgfra*/*Pdgfrb* for generating preadipocyte-specific KO models; in this study, we used tamoxifen-inducible *Pdgfrb-Cre* mice. Although *Pdgfrb-Cre* can also target myofibroblasts in various tissues, we confirmed specific PAK4 deletion in preadipocytes from SVCs of *Pak4*-KO mice, indicating that our model is sufficient for our hypothesis. Moreover, we focused only on male mice, as female hormones, especially estrogen, can impact adipogenesis^39^. Future research should include female mice. A notable strength of our study is the validation of findings from in vitro cultures and animal data in primary SVCs isolated from human fat pads. In summary, combined with our recent discovery that PAK4 suppresses lipolysis in adipocytes^23^, we propose that inhibiting PAK4 could be a promising therapeutic strategy for obesity.

## Supplementary information

Supplementary Information

## References

[1] Tang, Q. Q. & Lane, M. D. Adipogenesis: from stem cell to adipocyte. *Annu Rev. Biochem* **81**, 715–736 (2012). 22463691 10.1146/annurev-biochem-052110-115718

[2] Guo, L., Li, X. & Tang, Q. Q. Transcriptional regulation of adipocyte differentiation: a central role for CCAAT/enhancer-binding protein (C/EBP) beta. *J. Biol. Chem.* **290**, 755–761 (2015). 25451943 10.1074/jbc.R114.619957 PMC4294498

[3] Farmer, S. R. Regulation of PPARγ activity during adipogenesis. *Int J. Obes.* **29**, S13–S16 (2005). 10.1038/sj.ijo.0802907 15711576

[4] Student, A. K., Hsu, R. Y. & Lane, M. D. Induction of fatty acid synthetase synthesis in differentiating 3T3-L1 preadipocytes. *J. Biol. Chem.* **255**, 4745–4750 (1980). 7372608

[5] Tang, Q. Q., Otto, T. C. & Lane, M. D. Mitotic clonal expansion: a synchronous process required for adipogenesis. *Proc. Natl Acad. Sci. USA* **100**, 44–49 (2003). 12502791 10.1073/pnas.0137044100 PMC140878

[6] Kowenz-Leutz, E., Twamley, G., Ansieau, S. & Leutz, A. Novel mechanism of C/EBP beta (NF-M) transcriptional control: activation through derepression. *Genes Dev.* **8**, 2781–2791 (1994). 7958933 10.1101/gad.8.22.2781

[7] Tang, Q. Q. et al. Sequential phosphorylation of CCAAT enhancer-binding protein β by MAPK and glycogen synthase kinase 3β is required for adipogenesis. *Proc. Natl Acad. Sci. USA* **102**, 9766–9771 (2005). 15985551 10.1073/pnas.0503891102 PMC1175002

[8] Chinery, R., Brockman, J. A., Dransfield, D. T. & Coffey, R. J. Antioxidant-induced nuclear translocation of CCAAT/enhancer-binding protein β. A critical role for protein kinase A-mediated phosphorylation of Ser299. *J. Biol. Chem.* **272**, 30356–30361 (1997). 9374525 10.1074/jbc.272.48.30356

[9] Steinmann, S., Coulibaly, A., Ohnheiser, J., Jakobs, A. & Klempnauer, K. H. Interaction and cooperation of the CCAAT-box enhancer-binding protein β (C/EBPβ) with the homeodomain-interacting protein kinase 2 (Hipk2). *J. Biol. Chem.* **288**, 22257–22269 (2013). 23782693 10.1074/jbc.M113.487769 PMC3829317

[10] Atwood, A. A. & Sealy, L. Regulation of C/EBPβ1 by Ras in mammary epithelial cells and the role of C/EBPβ1 in oncogene-induced senescence. *Oncogene* **29**, 6004–6015 (2010). 20818427 10.1038/onc.2010.336 PMC2978746

[11] Zhao, X., Zhuang, S., Chen, Y., Boss, G. R. & Pilz, R. B. Cyclic GMP-dependent protein kinase regulates CCAAT enhancer-binding protein β functions through inhibition of glycogen synthase kinase-3. *J. Biol. Chem.* **280**, 32683–32692 (2005). 16055922 10.1074/jbc.M505486200

[12] Buck, M. & Chojkier, M. C/EBPβ phosphorylation rescues macrophage dysfunction and apoptosis induced by anthrax lethal toxin. *Am. J. Physiol. Cell Physiol.* **293**, C1788–C1796 (2007). 17855774 10.1152/ajpcell.00141.2007

[13] Wegner, M., Cao, Z. & Rosenfeld, M. G. Calcium-regulated phosphorylation within the leucine zipper of C/EBPβ. *Science* **256**, 370–373 (1992). 1314426 10.1126/science.256.5055.370

[14] Won, S. Y., Park, J. J., Shin, E. Y. & Kim, E. G. PAK4 signaling in health and disease: defining the PAK4–CREB axis. *Exp. Mol. Med* **51**, 1–9 (2019). 30755582 10.1038/s12276-018-0204-0 PMC6372590

[15] Costa, T. D. F. et al. PAK4 suppresses RELB to prevent senescence-like growth arrest in breast cancer. *Nat. Commun.* **10**, 3589 (2019). 31399573 10.1038/s41467-019-11510-4 PMC6689091

[16] Cai, S. et al. Overexpression of p21-activated kinase 4 is associated with poor prognosis in non-small cell lung cancer and promotes migration and invasion. *J. Exp. Clin. Cancer Res.* **34**, 48 (2015). 25975262 10.1186/s13046-015-0165-2 PMC4443662

[17] Park, M. H. et al. p21-Activated kinase 4 promotes prostate cancer progression through CREB. *Oncogene* **32**, 2475–2482 (2013). 22710715 10.1038/onc.2012.255

[18] Mao, Y. et al. p21-activated kinase 4 phosphorylates peroxisome proliferator-activated receptor γ and suppresses skeletal muscle regeneration. *J. Cachexia Sarcopenia Muscle* **12**, 1776–1788 (2021). 34431242 10.1002/jcsm.12774 PMC8718036

[19] Yu, H. C. et al. p21-activated kinase 4 and ischemic acute kidney injury in mice and humans. *J. Am. Soc. Nephrol.* **36**, 1264–1277 (2025). 40019790 10.1681/ASN.0000000649 PMC12187246

[20] Mao, Y. et al. p21-activated kinase 4 inhibition protects against liver ischemia/reperfusion injury: role of nuclear factor erythroid 2-related factor 2 phosphorylation. *Hepatology* **76**, 345–356 (2022). 35108418 10.1002/hep.32384

[21] Shi, M. Y. et al. p21-activated kinase 4 suppresses fatty acid β-oxidation and ketogenesis by phosphorylating NCoR1. *Nat. Commun.* **14**, 4987 (2023). 37591884 10.1038/s41467-023-40597-z PMC10435519

[22] Wu, D. et al. PAK4 phosphorylates and inhibits AMPKα to control glucose uptake. *Nat. Commun.* **15**, 6858 (2024). 39127697 10.1038/s41467-024-51240-w PMC11316743

[23] Yu, H. C. et al. p21-activated kinase 4 counteracts PKA-dependent lipolysis by phosphorylating FABP4 and HSL. *Nat. Metab.* **6**, 94–112 (2024). 38216738 10.1038/s42255-023-00957-x

[24] Song, M. Y. et al. Adipose sirtuin 6 drives macrophage polarization toward M2 through IL-4 production and maintains systemic insulin sensitivity in mice and humans. *Exp. Mol. Med.* **51**, 1–10 (2019). 31113929 10.1038/s12276-019-0256-9 PMC6529411

[25] Baskaran, Y. et al. An in cellulo-derived structure of PAK4 in complex with its inhibitor Inka1. *Nat. Commun.* **6**, 8681 (2015). 26607847 10.1038/ncomms9681 PMC4674680

[26] Honda, R. et al. The structure of cyclin E1/CDK2: implications for CDK2 activation and CDK2-independent roles. *EMBO J.* **24**, 452–463 (2005). 15660127 10.1038/sj.emboj.7600554 PMC548659

[27] Siu, M. K. et al. p21-activated kinase 4 regulates ovarian cancer cell proliferation, migration, and invasion and contributes to poor prognosis in patients. *Proc. Natl Acad. Sci. USA* **107**, 18622–18627 (2010). 20926745 10.1073/pnas.0907481107 PMC2972956

[28] Resnitzky, D., Gossen, M., Bujard, H. & Reed, S. I. Acceleration of the G_1_/S phase transition by expression of cyclins D_1_ and E with an inducible system. *Mol. Cell Biol.* **14**, 1669–1679 (1994). 8114703 10.1128/mcb.14.3.1669-1679.1994 PMC358525

[29] Murray, B. W. et al. Small-molecule p21-activated kinase inhibitor PF-3758309 is a potent inhibitor of oncogenic signaling and tumor growth. *Proc. Natl Acad. Sci. USA* **107**, 9446–9451 (2010). 20439741 10.1073/pnas.0911863107 PMC2889050

[30] Koundrioukoff, S. et al. A direct interaction between proliferating cell nuclear antigen (PCNA) and Cdk2 targets PCNA-interacting proteins for phosphorylation. *J. Biol. Chem.* **275**, 22882–22887 (2000). 10930425 10.1074/jbc.M001850200

[31] Henderson, N. C. et al. Targeting of alphav integrin identifies a core molecular pathway that regulates fibrosis in several organs. *Nat. Med* **19**, 1617–1624 (2013). 24216753 10.1038/nm.3282 PMC3855865

[32] Keenan, S. M., Bellone, C. & Baldassare, J. J. Cyclin-dependent kinase 2 nucleocytoplasmic translocation is regulated by extracellular regulated kinase. *J. Biol. Chem.* **276**, 22404–22409 (2001). 11304535 10.1074/jbc.M100409200

[33] Sebastian, B., Kakizuka, A. & Hunter, T. Cdc25M2 activation of cyclin-dependent kinases by dephosphorylation of threonine-14 and tyrosine-15. *Proc. Natl Acad. Sci. USA* **90**, 3521–3524 (1993). 8475101 10.1073/pnas.90.8.3521 PMC46332

[34] Lents, N. H., Keenan, S. M., Bellone, C. & Baldassare, J. J. Stimulation of the Raf/MEK/ERK cascade is necessary and sufficient for activation and Thr-160 phosphorylation of a nuclear-targeted CDK2. *J. Biol. Chem.* **277**, 47469–47475 (2002). 12359725 10.1074/jbc.M207425200

[35] Vacurova, E. et al. Mitochondrially targeted tamoxifen alleviates markers of obesity and type 2 diabetes mellitus in mice. *Nat. Commun.* **13**, 1866 (2022). 35387987 10.1038/s41467-022-29486-z PMC8987092

[36] Chang, Y. H. et al. Cisplatin-impaired adipogenic differentiation of adipose mesenchymal stem cells. *Cell Transpl.* **26**, 1077–1087 (2017). 10.3727/096368917X694886 PMC5657753 28155807

[37] Arunachalam, S., Tirupathi Pichiah, P. B. & Achiraman, S. Doxorubicin treatment inhibits PPARγ and may induce lipotoxicity by mimicking a type 2 diabetes-like condition in rodent models. *FEBS Lett.* **587**, 105–110 (2013). 23219922 10.1016/j.febslet.2012.11.019

[38] Keshet, R. et al. c-Abl tyrosine kinase promotes adipocyte differentiation by targeting PPAR-gamma 2. *Proc. Natl Acad. Sci. USA* **111**, 16365–16370 (2014). 25368164 10.1073/pnas.1411086111 PMC4246298

[39] Saavedra-Pena, R. D. M., Taylor, N. & Rodeheffer, M. S. Insights of the role of estrogen in obesity from two models of ERα deletion. *J. Mol. Endocrinol.* **68**, 179–194 (2022). 35244608 10.1530/JME-21-0260 PMC10173145

