# Supplementary material for: PAK4 phosphorylates cyclin-dependent kinase 2 to promote the G1/S transition during adipogenesis
Source: Exp Mol Med. 2025 Sep 30;57(9):2121–32. doi: 10.1038/s12276-025-01525-x (PMC12508475; doi:10.1038/s12276-025-01525-x)
Supplement: Supplementary file 1 — Supplementary Information [file 12276_2025_1525_MOESM1_ESM.pdf]

Supplementary Figures

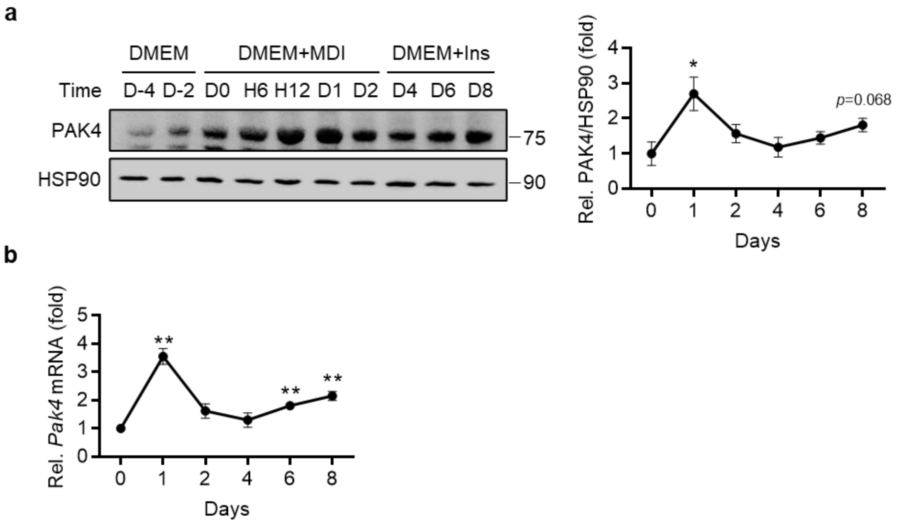

Supplementary Figure 1. PAK4 expression changes during various stages of adipogenesis in 3T3-L1 cells. a. Protein levels of PAK4 in 3T3-L1 cells induced to differentiate with MDI or insulin (Ins, 10 μg/ml) for the specified time periods were analyzed by Western blotting, with protein density quantified ($n = 4$). **b**. Quantitative was performed to assess *Pak4* mRNA levels following MDI treatment at the indicated time points ($n = 4$). Values are mean ± SD. *, $p < 0.05$.

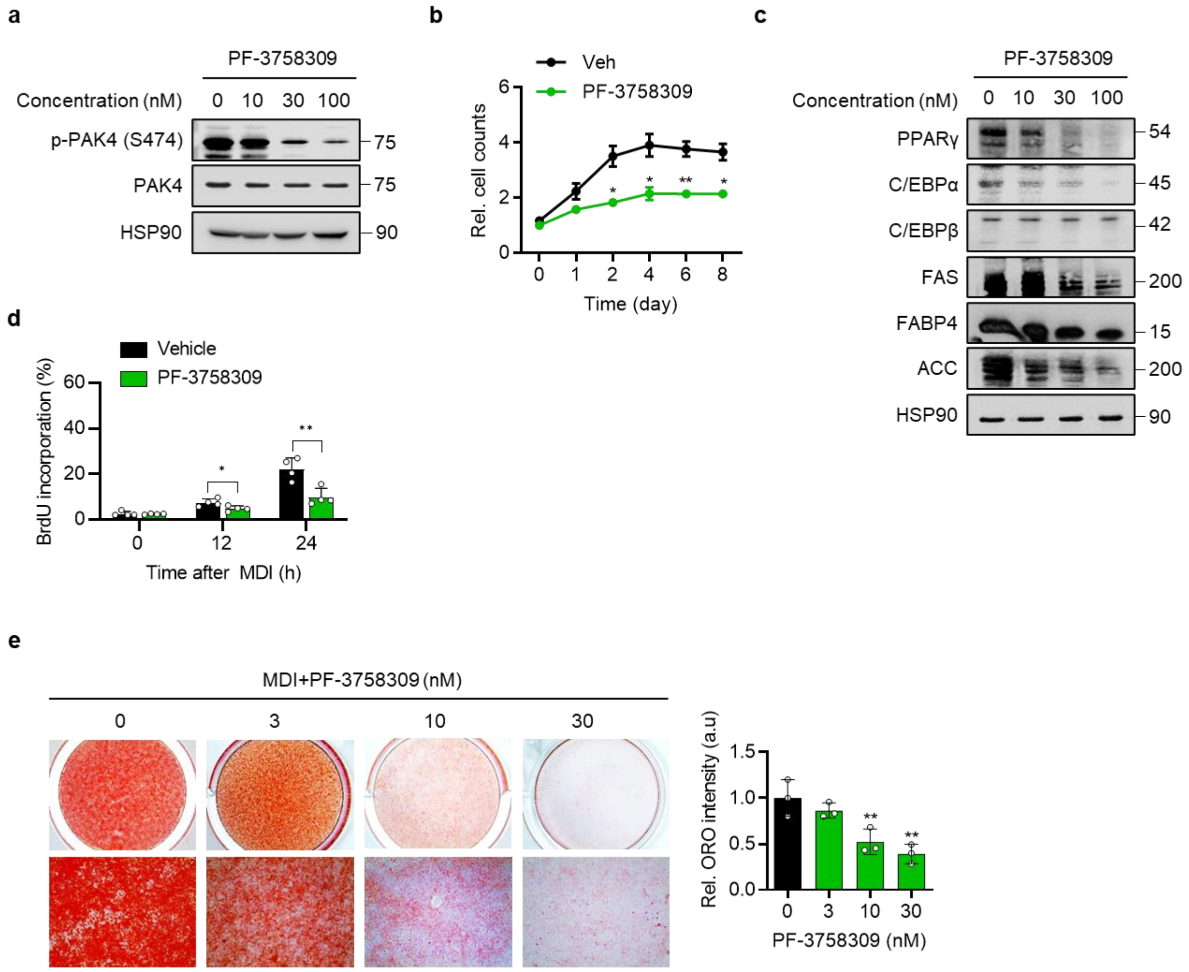

Supplementary Figure 2. Inhibition of adipogenesis by the PAK4 inhibitor PF-3758309. **a**-**c**. 3T3-L1 preadipocytes were treated with various concentrations of PF-3758309 for 24 h (a) or 8 days (c) or 10 nM PF-3758309 for different time periods (b), and the effects on PAK4 suppression, cell proliferation, and adipogenesis marker protein levels were evaluated. **d**. 3T3-L1 preadipocytes were treated with MDI for 12 or 24 h with or without 10 nM PF-3758309, and the percentage of BrdU incorporation was measured (*n* = 4) was determined. **e**. After 8 days of differentiation, representative images from each group were captured following Oil Red O staining to assess lipid accumulation. Values are mean ± SD. *, $p < 0.05$ and **, $p < 0.01$.

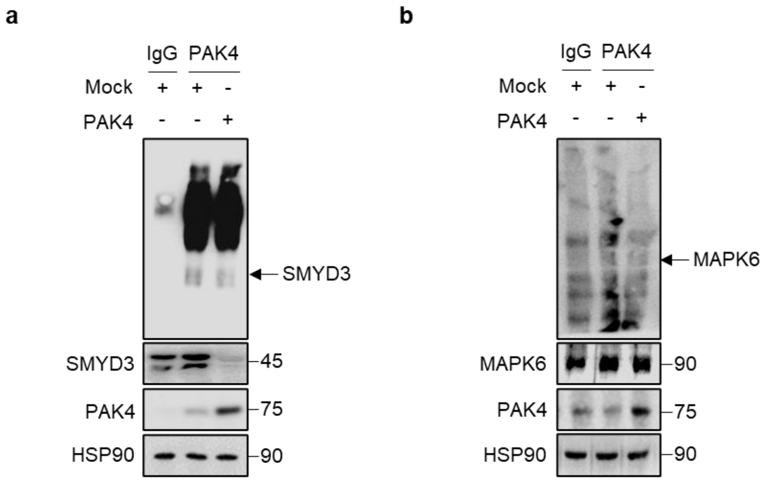

Supplementary Figure 3. No interaction between PAK4 and either SMYD3 or MAPK5.

a. **b**. Following transfection of 3T3-L1 preadipocytes as indicated, co-IP was conducted to assess the physical interactions between PAK4 and SMYD3 (a) and between PAK4 and MAPK6 (b).
